# Supplementary material for: Contribution of Hydrolysis and Drying Conditions to Whey Protein Hydrolysate Characteristics and In Vitro Antioxidative Properties
Source: Antioxidants (Basel). 2022 Feb 16;11(2):399. doi: 10.3390/antiox11020399 (PMC8869527; doi:10.3390/antiox11020399)
Supplement: Supplementary file 1 [file antioxidants-11-00399-s001.zip › antioxidants-1586816-supplementary.pdf]

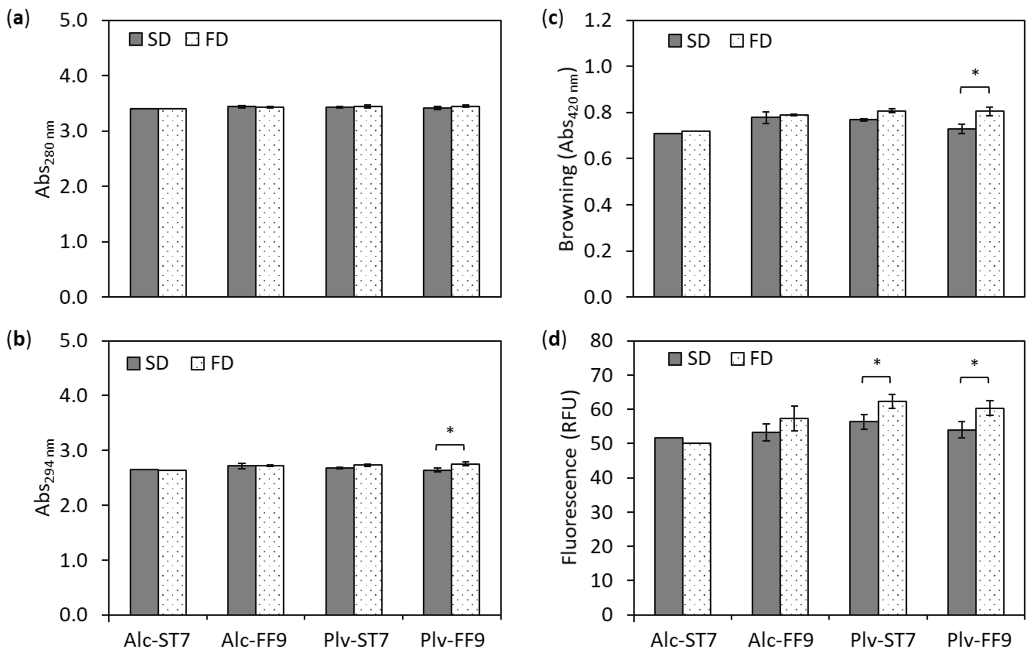

Figure S1: Effect of spray- (SD) and freeze-drying (FD) on non-fluorescence Maillard reaction intermediate products measured at (**a**) absorbance (Abs) 280 nm and (**b**) Abs 294 nm, (**c**) browning of late Maillard reaction products and (d) fluorescence of advanced Maillard reaction products expressed as relative fluorescence units (RFU) of whey protein hydrolysates prepared using Alcalase® (Alc) and Prolyve® (Plv) obtained under pH-stat at pH 7.0 (ST7) and free-fall pH 9.0 (FF9). Results represent mean ± SD (n = 3). * denotes significant difference at $p <$ 0.05.

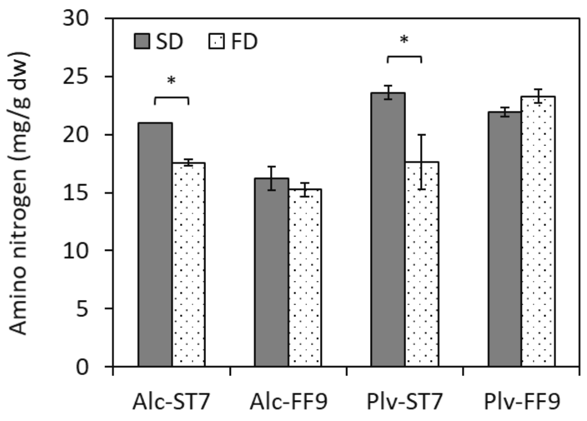

Figure S2: Effect of spray- (SD) and freeze-drying (FD) on amino nitrogen contents of whey protein hydrolysates prepared using Alcalase® (Alc) and Prolyve® (Plv) obtained under pH-stat at pH 7.0 (ST7) and free-fall pH 9.0 (FF9). Results represent mean ± SD (n = 3), where * denotes significant difference at $p < 0.05$.

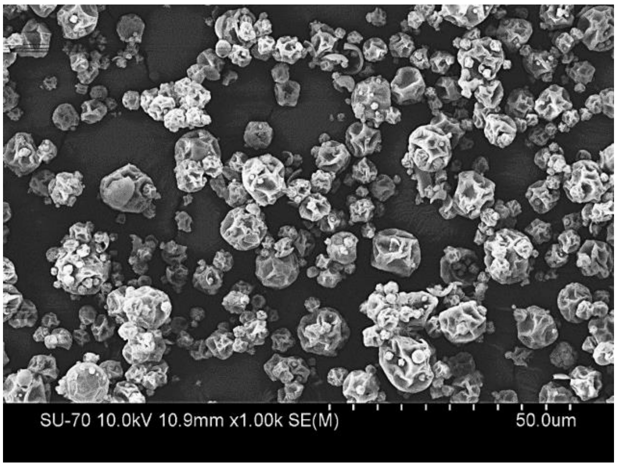

Figure S3: Scanning electron micrograph of whey protein concentrate (WPC80) powder used as a substrate for the generation of whey protein hydrolysates in the present study.
